# Uncertainty in IoT for Smart Healthcare: Challenges, and Opportunities

**DOI:** 10.1007/978-3-030-51517-1_19

**Published:** 2020-05-31

**Authors:** Anis Tissaoui, Malak Saidi

**Affiliations:** 8 Digital Research Centre of Sfax, Sfax, Tunisia; 9 Institut Mines-Télécom, CNRS, Paris, France; 10 Université de Sherbrooke, Sherbrooke, QC Canada; 11 Digital Research Centre of Sfax, Sfax, Tunisia; 12 University of Sfax, Sfax, Tunisia; VPNC Lab, FSJEG, University of Jendouba, Jendouba, Tunisia

**Keywords:** Uncertainty, Internet of Things, Smart Healthcare

## Abstract

According to Knight, uncertainty signifies deviations from the expected states, which prevent us from the use of any probability for the determination of a result for a given action or decision [1]. This paper describes the phenomenon of uncertainty in the face of technological megatrends and challenges associated with them. The article focuses on the analysis of the uncertainty in one of the most important technology trends – the Internet of Things (IoT) – on the example of Healthcare. The right decisions are not always equivalent to good results. Sometimes, the decision taken in accordance with general rules brings worse results than the one who breaks them. Such a situation is possible as a result of the uncertainty accompanying the predictions of the future. In this article the concept of the IoT is treated as a big, complex, dynamic system with specific characteristics, dimensions. structures and behaviors. The aim of the article is to analyze the factors that may determine the uncertainty and ambiguity of such systems in the context of the development of Healthcare, and recommendations are made for future research directions.

## Introduction

The basic idea of the Internet of Things is the pervasive presence around us of a variety of things or objects – such as Radio-Frequency IDentification (RFID) tags, sensors, actuators, mobile phones, etc. – which, through unique addressing schemes, are able to interact with each other and cooperate with their neighbors to reach common goals [7].

This The advent of the Internet of Things is changing people’s lives and their integration with the surrounding environment. It is estimated that the number of connected IoT devices will outgrow the world population and increase to 50 billion by end 2020 [2]. The technical evolution of IoT also stimulates the development of smart homes. It not only makes people’s daily living more convenient, but also can contribute solutions for challenges in healthcare system [3, 4].

**Fig. 1.**
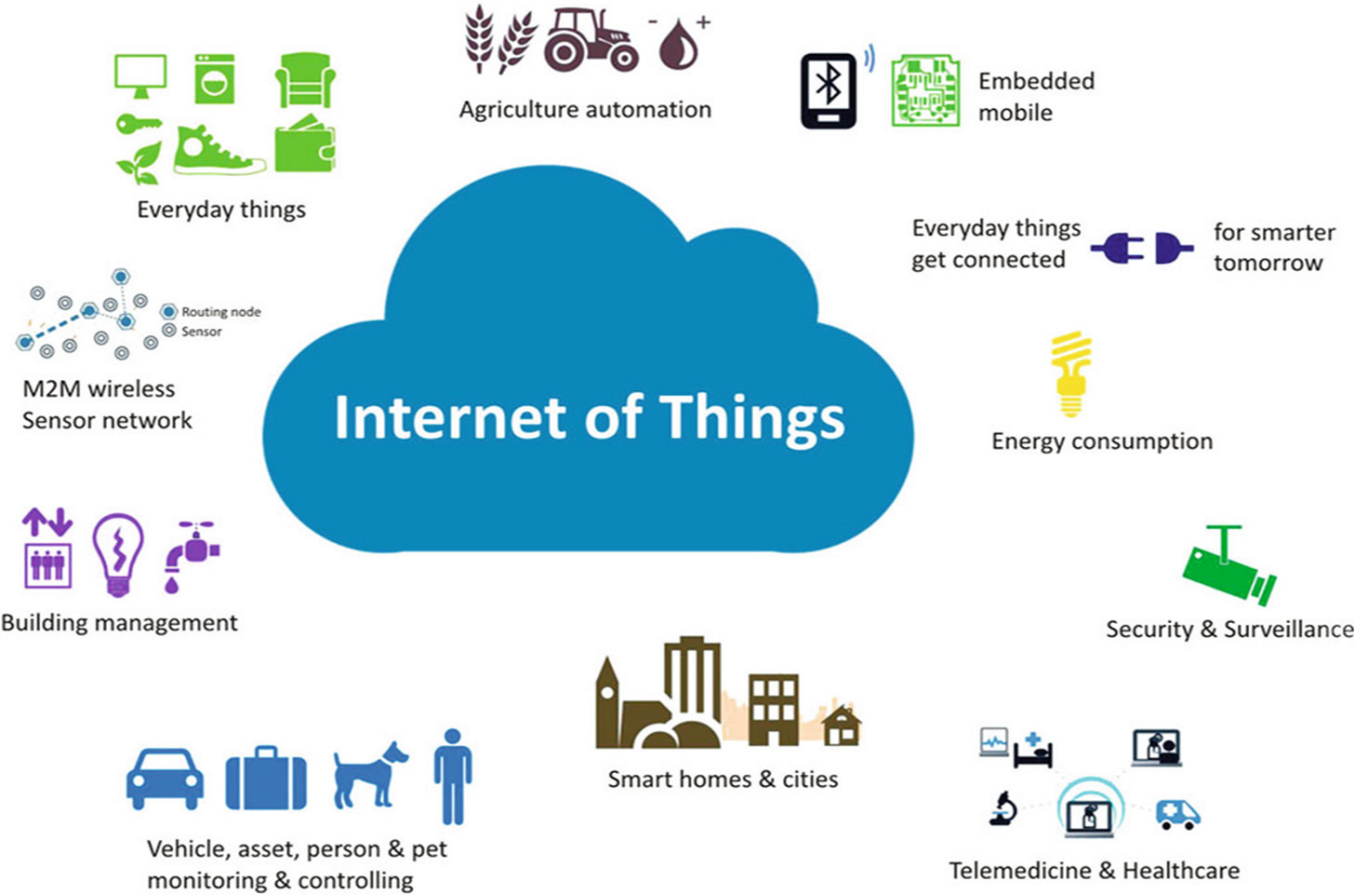
Figure showing the Internet of Things application areas

Key fields and applications (This can be portrayed in Fig. 1) for applying IoT solutions encompass [5]: smart cities, smart power networks, smart transport and smart buildings (intelligent solutions for living). This list also includes smart health care, one of the main subjects of this article.

The IoT field is a long-range technological area which presents great opportunities for development. Since the Internet of Things is a relatively new domain it carries a high level of uncertainty, both in relation to IoT technologies as well as to the aspects which are (or will be) correlated to this field, such as social, economic, technological, legal, etc. aspects [6]. Various characteristics, structures and behaviors of the IoT system are beyond areas which, so far, have been observed and are within verified knowledge, which creates significant uncertainty regarding future situations.

Healthcare is undergoing a rapid transformation from traditional hospital and specialist focused approach to a distributed patient-centric approach. The IoT applications are obscurity essential in remodeling lives of individuals than in health care. Internet of Things indicates to physical devices, like a measuring system, weight scale and patients very important watching devices (glucose, force per unit area, vital sign, and activity watching, etc.) connect with the web and transforms data from the physical to the digital world.

At present, healthcare widely uses IoT are continually evolving to accommodate the needs of future intelligent healthcare applications. This will place complex demands in terms of heterogeneity of devices, scalability, wide scale use of wireless data transfer technology, optimum energy use, data management, privacy protection bandwidth, data rate and latency, among other factors.

In the field of healthcare, various types of uncertainty are distinguished in the assessment of innovative programmes: parameter uncertainty, structural uncertainty, methodological uncertainty, variability, heterogeneity and decision uncertainty [12]. The article addresses the phenomenon of uncertainty occurring in large, developing systems namely the Internet of Things through the use of the example of the smart Health care.

## The IoT in Health Care

There is a great potential for applying IoT technology across all sectors including both industrial and public to improve operation efficiency, reduce cost, and provide better service. In the healthcare, IoT plays a very important role in various applications. This criterion is divided into three phases, such as clinical care, remote monitoring and context awareness. During data collection, the risks of human error are reduced by means of automatic medical data collection method. This will improve the quality of the diagnosis and reduce the risk of human errors, who are involved in the collection or transmission of false information which is dangerous for the patients’ health. There have been efforts for reviewing healthcare with different aspects.

This Arcadius et al. explained about the WBAN^1^ based on IoT for the healthcare applications as it communicated with the individual to individual and the individual to things [13]. According to the author, the IoT was made to be a part of the overall Internet of the future and several technologies were used in the IoT such as communication solutions, tracking technology, wired and wireless sensor identification etc.

Jara et al. described the interconnection framework for m- healthcare application based on IoT [14]. The process of communication and the information access process had a personalized health end to end framework. The personalized data was complex and was found to be in an incomplete manner. So, the authors introduced interconnection framework for m-healthcare applications based on IoT. It made continuous and real-time vital sign monitoring system which introduced technological innovations for the health monitoring of patient’s devices by means of Internet system.

Qi et al. [7] explored various applications of IoT in smart healthcare from different perspectives (i.e., Blood pressure monitoring, monitoring of oxygen saturation, heartbeat monitoring etc.). Islam et al. focused on IoT-based healthcare technologies and present architecture for healthcare network and platforms which support access to the IoT backbone and enable medical data reception and data transmission. Secondly, the paper delivers detailed research events and how the IoT can address chronic disease supervision, pediatric, care of elderly and fitness management [8].

Catarinucci et al. modeled a smart healthcare system based on IoT aware architecture [15]. The authors introduced the IoT aware architecture for automatic monitoring and tracking of patient’s biomedical information. They also proposed smart hospital system, with enabling technologies, especially for wireless networks and smart mobile to enable network infrastructure. Thus, it provided highly efficient real-time monitoring of patient’s biomedical information. Furthermore, privacy was an open issue in this system; Baker et al. [9] presented a new model for future smart healthcare systems, which can be used for both special (i.e., special condition monitoring) and general systems. Nowadays, all over the world, there are many people whose health might suffer due to lack of effective healthcare monitoring. Elderly, children or chronically ill people needed to be examined almost daily. Remote monitoring is an important paradigm for many real- world applications.

Context-awareness is a major criterion in the healthcare IoT applications. As it has the ability to find the patient’s condition and the environment where the patient was located it will greatly assist the healthcare professionals to understand the variations that can influence the health status of these patients. In addition to, the change of physical state of the patient may increase the percentage of its vulnerabilities to diseases and be a cause for his/her health deterioration [10].

Mahmoud et al. [11] focused on different IoT-based healthcare systems for Wireless Body Area Network (WBAN) that can enable smart healthcare data reception and data transmission. the author presented a detailed of resource management, power, energy, security and privacy related to IoT-based smart healthcare.

## Characteristics of the Phenomenon of Uncertainty

### Defining Uncertainty

The concept of uncertainty encompasses multiple aspects and meanings. The widely spread use of the concept of uncertainty throughout various scientific disciplines, as well as in everyday language, has caused it to acquire many definitions.

According to F.H. Knight, uncertainty signifies deviations from the expected states, which prevent us from the use of any probability for the determination of a result for a given action or decision [1]. E. Ostrowska follows F. Knight with the measurable and immeasurable uncertainty theory which defines the former as risk and the latter as immeasurable uncertainty in its strict sense.

According to A.H. Willet uncertainty concerns changes which are difficult to estimate, or events whose probability cannot be predicted because the amount of available information is too limited [16]. A. Jøsang [17] proves that uncertainty in its strict sense can be measured using subjective logic. Subjective logic is a type of probabilistic logic that allows probability values to be expressed with degrees of uncertainty. The idea of subjective logic is to extend probabilistic logic by also expressing uncertainty about the probability values themselves, meaning that it is possible to reason with argument models in the presence of uncertain or incomplete evidence.

### Types of Uncertainty in the Field of Healthcare

Depending on the contexts being studied in the field of healthcare, various types of uncertainty are distinguished in the assessment of innovative programmes: parameter uncertainty, structural uncertainty, methodological uncertainty, variability, heterogeneity and decision uncertainty [12].

Structural uncertainty refers to uncertainty surrounding the structure of a decision model. Variability relates to the fact that individuals are unique and therefore vary in their outcomes, which may partly be explained by individual characteristics [19]. Parameter uncertainty relates to the fact that the true value of a parameter is not known [18]. In practice, it mostly refers to imprecise estimates and standard errors surrounding a mean value, which corresponds to measurement error. Decision uncertainty is the umbrella term for all uncertainty surrounding a decision, and can be caused by any other type of uncertainty [12]. Methodological uncertainty can be defined as disparities in the choice of analytic methods that underpin an assessment [18].

### Sources of Uncertainty in IoT Systems

Several of other factors could influence the occurrence of uncertainty in IoT. Key characteristics of IoT which influence uncertainty include:

- *heterogeneity of devices or Interoperability*: Interoperability plays an important role in smart healthcare, providing connectivity between different devices using different communication technologies. Interoperability between different devices in different domains is a key limitation for IoT success due to lack of universal standards. the large number of devices used means high diversity in their calculation and communication capabilities.
- *Resources constraints (energy and computational and storage capabilities)*: the issue of power use is crucial. IoT devices used for healthcare are connected with a collection of sensors. A continuous source of energy is required to drive these devices, which presents a severe challenge in term of cost and battery life. Their computational and storage capabilities do not allow complex operations support (e.g. cryptographic operations, etc.).
- *privacy protection*: The security protection is not about encrypting/decrypting user data, but about how a user in a heath community can use trust information to filter out untrustworthy input when gathering health information to enhance IoT health security. Due to constrained nature of IoT device (limited processing and battery life) it is difficult to implement complex security protocols and algorithms. This leads to numerous attacks and threats in term of security and privacy.
- *scalability (connectivity in IoT)*: connectivity of a growing number of devices being used every day. A smart healthcare network consists of billions of devices. can succeed only if it can provide capabilities of sensing to produce important information.
- *data management*: In smart healthcare, billions of devices are connected, which can produce a huge amount of data and information for analysis. in IoT it will be crucial to utilize appropriate data models and semantic descriptions of their content, appropriate language and format.
- *Network*: Intermittent loss of connection in the IoT is fairly frequent. In fact, IoT is seen as an IP network with more constraints and a higher ratio of packet loss problems connected with overcoming this issue are related to transfer speeds and delays in delivery of data;
- *Quality of Services (QoS)*: the quality of services is an important parameter used in the healthcare services which is a highly time-sensitive system. Numerous challenges exist to meet the quality requirements of IoT-based applications in terms of energy efficiency, sensing data quality, network resource consumption, and latency. The quality of body sensors determines the accuracy and sensitivity measurements provided by a sensor.

### Causes of Uncertainty in IoT Systems

Uncertainty is one of the key problems for most IoT systems based on RFID (Radio Frequency IDentification) technology. Listed below are causes of uncertainty relating to the following fields [20]:

- *Inconsistent data (unbounded data, data conflict)*: RFID tags can be read using various readers at the same time therefore it is possible to get inconsistent data about the exact location of tags;
- *Incomplete data (Noisy data, data loss)*: tagged objects might be stolen or forged and generate fake data.
- *Ambiguity Data (plausibility, imprecision)*: sometimes radio frequencies might cause data to be reflected in reading areas, so RFID readers might read those reflections;
- *Missing readings*: tag collisions, tag detuning, metal/liquid effect, tag misalignment;
- *Redundant data*: captured data may contain significant amounts of additional information;

## Findings and Recommendations

### Research Challenges

- How to guarantee connectivity of massive IoT devices in a wide range during high mobility?
- How to guarantee resource management in highly dense network?
- How to utilize power/energy of IoT devices?
- How to extend IoT devices battery life?
- Incorporating devices for retailer locked-in services.
- Secure integration and deployment of services (cloud-based) at both device and network levels.
- Early detection of both outsider and insider threats.
- Standardized security solutions without delaying data integrity.

### Major Requirements

- adaptation of trust management mechanisms, similarly to what was already adopted for P2P and grid systems and technical security policies;
- Identification of vulnerabilities at a various level in the network. which work as entry points for numerous attacks.
- adaptation of trust relationships on the following levels:
  - IoT entities;
  - data perception (sensor sensibility, preciseness, security, reliability, persistence, data collection efficiency);
  - privacy preservation (user data and personal information);
  - data fusion and mining;
  - data transmission and communication;
  - quality of IoT services;
  - acceptance of shared standards to cope with the diversity of devices and applications;
- creation of simulations and models of uncertainty phenomena;

## Footnotes

1 Wireless Body Area Network.
